# PP2A-Like Protein Phosphatase (*Sit4*) Regulatory Subunits, *Sap155* and *Sap190*, Regulate *Candida albicans*’ Cell Growth, Morphogenesis, and Virulence

**DOI:** 10.3389/fmicb.2019.02943

**Published:** 2019-12-20

**Authors:** Qi Han, Chaoying Pan, Yueqing Wang, Linpeng Zhao, Yue Wang, Jianli Sang

**Affiliations:** ^1^School of Life Sciences, Beijing University of Chinese Medicine, Beijing, China; ^2^Key Laboratory of Cell Proliferation and Regulation Biology, College of Life Sciences, Beijing Normal University, Beijing, China; ^3^Institute of Molecular and Cell Biology, Agency for Science, Technology and Research, Singapore, Singapore; ^4^Depatment of Biochemistry, Yong Loo Lin School of Medicine, National University of Singapore, Singapore, Singapore

**Keywords:** *SAP155*, *SAP190*, *SIT4*, *Candida albicans*, morphogenesis, virulence

## Abstract

PP2A-like phosphatases share high homology with PP2A enzymes and are composed of a catalytic subunit and a regulatory subunit. In *Candida albicans*, the PP2A-like catalytic subunit *SIT4* regulates cell growth, morphogenesis, and virulence. However, the functions of its regulatory subunits remain unclear. Here, by homology analysis and co-IP experiments, we identified two regulatory subunits of *SIT4* in *C. albicans*, *SAP155* (orf19.642) and *SAP190* (orf19.5160). We constructed *sit4*Δ/Δ, *sap155*Δ/Δ, *sap190*Δ/Δ, and *sap155*Δ/Δ *sap190*Δ/Δ mutants and found that deleting *SAP155* had no apparent phenotypic consequence, while deleting *SAP190* caused slow growth, hypersensitivity to cell wall stress, abnormal morphogenesis in response to serum or genotoxic stress (HU and MMS), less damage to macrophages, and attenuated virulence in mice. However, deleting both *SAP155* and *SAP190* caused significantly stronger defects, which was similar to deleting *SIT4*. Together, our results suggest that *SAP190* is required for the function of *SIT4* and that *SAP155* can partially compensate for the loss of *SAP190* in *C. albicans*. Given the vital role of these regulatory subunits of *SIT4* in *C. albicans* physiology and virulence, they could serve as potential antifungal targets.

## Introduction

*Candida albicans* (Ca) is a commensal organism of the oral cavity, gastrointestinal tract, and vagina (Arendrup, 2013; Hebecker et al., 2014). When the host immune system is compromised, such as under conditions of long-term antibiotic treatment, immunodeficiency, or chemotherapy, *C. albicans* can cause mucocutaneous and life-threatening disseminated infections (Romani, 2011; Goulart et al., 2018). According to statistics, *C. albicans* is the fourth most common cause of hospital-acquired systemic infections with a crude mortality rate of more than 50% in the United States (Lai et al., 2008; Pfaller and Diekema, 2010). *C. albicans* can grow as several cell types, including yeast, pseudohyphae, and true hyphae (Sudbery et al., 2004). Yeast form helps its spread, while hyphae have strong ability of tissue adhesion and invasion (Berman and Sudbery, 2002; Zhu and Filler, 2010). Furthermore, hyphae can avoid recognition and phagocytosis by host macrophages and neutrophils, thus enabling it to escape from the killing of the host immune system (Erwig and Gow, 2016). The transformation between different cell types is closely related to *C. albicans* pathogenicity (Lo et al., 1997; Saville et al., 2003), suggesting that the identification of proteins involved in morphogenesis may provide new targets for developing antifungal agents.

Reversible protein phosphorylation plays a crucial role in the control of nearly all cellular processes, and dephosphorylation is equally important to phosphorylation. Most phosphorylation events in eukaryotes involve the transfer of phosphate to serine (Ser) or threonine (Thr) residues. Removal of the phosphate is catalyzed by Ser/Thr protein phosphatases. According to the enzymological criteria, Ser/Thr protein phosphatases can be classified into two groups: type 1 (PP1) and type 2 (PP2); PP2 phosphatases can be further classified into several groups based on the requirement for metal ions: PP2A and PP2A-like enzymes do not require metal ions, PP2B is activated by calcium, and 2C is Mg^2+^ dependent (Arino et al., 2011; Albataineh and Kadosh, 2016). There are three PP2A-like phosphatases in fungi: *Sit4*, Pph3, and Ppg1 (Albataineh and Kadosh, 2016). In *Saccharomyces cerevisiae*, *Sit4* plays a critical role in cell growth, proliferation, and the regulation of the Pkc1-MAPK and Tor signaling pathways (Ronne et al., 1991; Sutton et al., 1991; Angeles et al., 2002; Rohde et al., 2004). Four regulatory subunits of *Sit4* has been identified, and they are named *Sit4* association proteins (SAPs) and divided into two groups based on sequence similarity, the *SAP4*/*SAP155* group and the *SAP185*/*SAP190* group (Luke et al., 1996). Studies have shown that the SAPs have diverse functions, such as the regulation of cell growth, K^+^ efflux, and drug resistance (Luke et al., 1996; Manlandro et al., 2005; Miranda et al., 2010). In *C. albicans*, *Sit4* has been identified as the catalytic subunit of PP2A-like protein phosphatase, and deletion of *SIT4* causes a significant reduction in growth rate, morphogenesis, and virulence in mice (Lee et al., 2004; Noble et al., 2010). However, the functions of its regulatory subunits remain unclear. According to a search of *C. albicans* genome database^1^, we identified two regulatory subunits of *Sit4*, orf19.642, and orf19.5160.

In this study, we constructed *sit4*Δ/Δ, *sap155*Δ/Δ, *sap190*Δ/Δ, and *sap155*Δ/Δ *sap190*Δ/Δ mutants in *C. albicans* SC5314 background and conducted comprehensive phenotypic characterizations and comparisons. We found that *Sap190* is the main regulatory subunit of *Sit4* that plays critical roles in cell growth, cell wall integrity, hyphal morphogenesis, and virulence. Sap155 is a redundant regulatory subunit, but it is functional and can partially compensate for the absence of *Sap190*.

## Materials and Methods

### Strains and Growth Conditions

The *Candida albicans* strains used in this study are listed in Table 1. *C. albicans* was routinely grown at 30^°^C in YPD medium (1% yeast extract, 2% peptone, and 2% glucose). For growth on plates, 2% agar was added to the medium. To select for nourseothricin-resistant transformants, 200 μg/mL of nourseothricin (Werner Bioagents, Jena, Germany) was added to the YPD agar plates (YPD-Nou plates). To obtain nourseothricin-sensitive derivatives in which the *SAT1*-flipper was excised by FLP-mediated recombination, transformants were grown overnight in YCB–BSA medium (2.34% w/v yeast carbon base, 0.4% w/v bovine serum albumin, pH 4.0) to induce the *SAP2* promoter controlling *Ca*FLP expression, and then streak-inoculated onto YPD plates containing 25 μg/mL nourseothricin and incubated at 30^°^C at least 2 days.

**TABLE 1:** Candida albicans strains used in this study.

**Strain**	**Relevant genotype**	**Source**
SC5314	Wild type	Fonzi and Irwin, 1993
*Sit4*-Flag	*SIT4*/*SIT4-Flag-FRT*	This study
Sap155-GFP	*SAP155*/*SAP155-GFP-FRT*	This study
Sap155-Flag	*SAP155*/*SAP155-Flag-FRT*	This study
*Sap190*-GFP	*SAP190*/*SAP190-GFP-FRT*	This study
*Sit4*-Flag Sap155-GFP	*SIT4*/*SIT4-Flag-FRT SAP155*/*SAP155-GFP-FRT*	This study
*Sit4*-Flag *Sap190*-GFP	*SIT4*/*SIT4-Flag-FRT SAP190*/*SAP190-GFP-FRT*	This study
Sap155-Flag *Sap190*-GFP	*SAP155*/*SAP155-Flag-FRT SAP190*/*SAP190-GFP-FRT*	This study
*sit4*Δ/Δ	*sit4*Δ*:FRT*/*sit4*Δ*:FRT*	This study
*sap155*Δ/Δ	*sap155*Δ*:FRT*/*sap155*Δ*:FRT*	This study
*sap190*Δ/Δ	*sap190*Δ*:FRT*/*sap190*Δ*:FRT*	This study
*sap155*Δ/Δ *sap190*Δ/Δ	*sap155*Δ*:FRT*/*sap155*Δ*:FRT sap190*Δ*:FRT*/*sap190*Δ*:FRT*	This study
*SAP190/sap190*Δ	*SAP190*/*sap190*Δ*:FRT*	This study
*sap190*Δ/Δ + pAG6	*sap190*Δ*:FRT*/*sap190*Δ*:FRT SAT1*	This study
*sap190*Δ/Δ + *SAP190*	*sap190*Δ*:FRT*/*sap190*Δ*:FRT SAP190-SAT1*	This study

Hyphal growth was induced by supplementing YPD medium with 10% fetal calf serum or DMEM, and incubating at 37^°^C with shaking at 200 rpm, or steaking yeast cells onto Spider agar plates (1% w/v beef extract, 1% w/v mannitol, 0.2% w/v K_2_HPO_4_, and 2% w/v agar, pH 7.2) to incubate at 30^°^C for 7 days. Pseudohyphal growth was induced by supplementing YPD medium with 15 mM hydroxyurea or 0.02% methyl methanesulfonate (MMS), and incubating at 30^°^C with shaking at 200 rpm.

### Strain Construction

Gene deletion was done in *C. albicans* SC5314 using the *SAT*-flipper method as described previously (Reuss et al., 2004). Briefly, the *SAT1*-flipper cassette flanked by 60 bp of upstream and downstream sequences of the target gene was amplified by PCR. Then, the PCR products were transformed into SC5314 cells using the lithium acetate protocol. After transformation, cells were recovered by culturing in fresh YPD medium at 30^°^C for 4 h with shaking at 200 rpm before spreading onto YPD-Nou plates. Two round of the transformation were required to obtained homozygous deletion mutants. Genomic DNA and total RNA were isolated from selected transformants to verify the mutations by PCR and RT-PCR analysis.

The plasmid *pAG6* was constructed by Vylkova and Lorenz (2014), which is a *SAT1*-marked version of *CIp10* and used to integrate a gene into the *RP10* locus by linearizing with *Stu*I. We cloned *SAP190* into the *Kpn*I–*Xho*I sites of *pAG6*, then linearized the plasmid with *Stu*I, and transformed it into the *sap190*Δ/Δ mutant to obtain *SAP190* complemented strain.

To construct *SAT1*-marked version of GFP or Flag-tagging vectors, GFP or Flag gene sequence followed by the *URA3* terminator was inserted into the *Apa*I–*Xho*I sites of *pSFS1*. To tag protein with GFP or Flag at the C-terminus, the GFP or Flag-*SAT1*-flipper cassette flanked by 60 bp of the coding sequence 5′ to the stop codon (without the stop codon) and 60 bp of the non-coding sequence 3′ to the stop codon was amplified by PCR. The PCR products were transformed into appropriate strains. Correct tagging was verified by PCR and Western blotting analysis. The oligonucleotide primers used to construct deletion cassette and fusion protein are shown in Table 2.

**TABLE 2:** Primers used in this study.

**Name**	**Sequence (5′ to 3′)**	**Description**
*SIT4*-A	ACAATTATTCATATACTTCAGTT ACTATAAATTTGACAGAATACAT AAATACAGCAAATCGCTGG GTACCGGGCCCCCCTCGAG	To delete *SIT4*
*SIT4*-B	TTAGCATCATTAAGGGATTTGAAA AAAAAAGATATAAATATAAAAATC ATTCATCATTCGGGGCGAATTG GAGCTCCACCGCGG	To delete *SIT4*, and tag *SIT4* with Flag
SAP155-A	TCAAATCAAACTTTCAATAATA AGGAATCCTTCATCAAACATTA GACAATTTCAGCAACCGCTGGG TACCGGGCCCCCCTCGAG	To delete *SAP155*
SAP155-B	TAAATAAAGAAATAAATAAA ATCTTGAAATACAATTAAAAT CTTGAAATATACATGTAATGGG CGAATTGGAGCTCCACCGCGG	To delete *SAP155*, and tag *SAP155* with Flag or GFP
*SAP190*-A	TATTCCATCATTTTTTTTTG TTTTTGTTTTATTGTGTATTAA TAGCATTAATTATTTATAGCTGG GTACCGGGCCCCCCTCGAG	To delete *SAP190*
*SAP190*-B	CTCTCTATATATATCAAAGGGG AAACTATACATACTTATTAAAG AATATTTCTTCAATGTGGGCGAAT TGGAGCTCCACCGCGG	To delete *SAP190*, and tag *SAP190* with GFP
*SIT4*-Flag-A	GATGGTGACTTATCAGTCAAG AACAATGCCAACAAACAACAAAG AAGTGATTATTTTTTGGGGCCC GATTACAAGGATGACGAC	To tag *SIT4* with Flag
SAP155-Flag-A	GAAGACGAAGACATTGGAGA AACTAACAAATTAAAAAGAGTACC CACACATAATGATGAT*GGGC* *CCGATTACAAGGATGACGAC*	To tag *SAP155* with Flag.
SAP155-GFP-A	GAAGACGAAGACATTGGAGA AACTAACAAATTAAAAAGAGTA CCCACACATAATGATGATATGT CTAAAGGTGAAGAATTATTC	To tag *SAP155* with GFP.
*SAP190*-GFP-A	GATAGCTCAGACGAAGAGGAAA AACAAGACACAAAGCTTACAA GATCAGCAAGTAAAGGTATGTCT AAAGGTGAAGAATTATTC	To tag *SAP190* with GFP.
Re*SAP190*-A (*Kpn*I)	CGGGGTACCTAGTTGAAA GAATATTAATGGAAC	To clone *SAP190* into the *Kpn*I–*Xho*I sites of *pAG6*
Re*SAP190*-B (*Xho*I)	CCGCTCGAGGAATATCAA CCGGGATTATTTAAG	

### Growth Curves

Late-log phase *C. albicans* yeast cells were diluted to OD_600_ = 0.01 in 10 mL of YPD medium and were cultured at 30°C with shaking at 200 rpm. 100 μL of the culture was collected every 2 h, and OD_600_ was measured using a microplate reader. The experiment was performed in triplicate.

### Susceptibility Tests

*Candida albicans* cells grown to the late-log phase in YPD medium were harvested and washed twice with sterile water. The cell suspensions were 10-fold serially diluted to generate suspensions containing 10^6^ to 10^3^ cells/mL, and 5 μL of each dilution was spotted onto YPD plates containing the indicated concentrations of chemicals or drugs. Growth was assessed by incubating the plates at 30°C for the indicated time. All experiments were performed at least thrice.

### Fluorescence Microscopy

Log-phase *C. albicans* yeast cells were stained with 10 μg/mL DAPI to visualize nuclei. Cells were examined by differential interference contrast (DIC) and fluorescence microscopy.

### Co-immunoprecipitation (Co-IP) and Western Blotting (WB)

Co-IP and WB was performed as described previously by Han et al. (2019).

### Macrophage Cytotoxicity Assay

*Candida albicans* toxicity on macrophages was assessed by using a Cytotoxicity LDH Assay Kit-WST (Dojindo Molecular Technologies, Inc). RAW264.7 macrophages were seeded at 2.5 × 10^5^ cells per well of a 96-well tissue culture plate in phenol red-free DMEM and maintained for 6 h in a humidified incubator in 5% CO_2_ at 37^°^C. *C. albicans* cells were grown to the mid-log phase in YPD medium and washed twice with sterile PBS, and these cell suspensions were co-cultured with macrophages at a 3:1 ratio for 5 h. Supernatants were transferred into new plates and the absorbance at 490 nm was measured by a microplate reader. Cytotoxicity was calculated according to the average absorbance from each triplicate set of infected host cells relative to the maximum LDH release from lysed host cells following the manufacturer’s protocol. The experiment was performed in triplicates.

### Murine Model of Disseminated Candidiasis

Mid-log phase *C. albicans* yeast cells were washed twice and diluted to 5 × 10^6^ cells/mL with PBS. Ten female BALB/c mice per strain were injected via the tail vein with 200 μL of the cell suspension. The mice were monitored twice daily for survival for 20 days. To determine the organ fungal burden, five mice were infected with each strain as described above and sacrificed at 48 h after the injection to surgically remove the kidney. One kidney from each mouse was removed, weighed, and homogenized. The homogenate was serially diluted in PBS and spread onto YPD plates for counting colony forming units (CFUs) per gram of kidney. Another kidney was fixed with formaldehyde followed by 70% ethanol and then embedded in paraffin. Thin sections were cut and stained with periodic acid-Schiff staining for microscopic examination. Animal experiments were carried out in accordance to National Advisory Committee for Laboratory Animal Research Guidelines, and all procedures were approved by the IACUC of the Agency for Science, Technology and Research of Singapore.

### Statistical Analyses

In this study, all data are presented as mean ± SD based on results from at least 3 independent experiments. The results of the *in vitro* experiments were analyzed with Student’s *t*-test. The results of survival curves and fungal burdens were analyzed using Kaplan-Meier test and Mann-Whitney test, respectively. A *p* value less than 0.05 was considered statistically significant.

## Results

### CaSap155 or Ca*Sap190* Interacts With Ca*Sit4* in Co-IP Experiments

In the *C. albicans* genome database, orf19.642 and orf19.5160 are designated as the regulatory subunits of *Sit4*, and their amino-acid sequence homologies with ScSap4/cSap155/ScSap185/Sc*Sap190* are 23.3%/26.5%/23.8%/24.9% and 26.2%/26.5%/ 30.9%/35.0%, respectively (Supplementary Figure S1). Thus, we named them CaSap155 (orf19.642) and Ca*Sap190* (orf19.5160) in this study.

**FIGURE 1:**
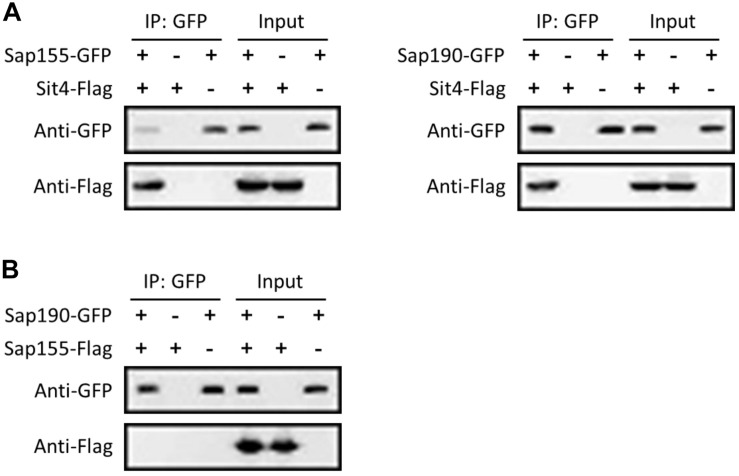
Ca*Sit4* interact with either CaSap155 or Ca*Sap190*. **(A)** Co-IP of *Sit4* with Sap155 and *Sap190*, and **(B)** co-IP of Sap155 with Sap190. Anti-GFP conjugated beads were used for pull-down from log-phase yeast cell lysates, and the pull-down products were probed with anti-GFP or anti-Flag in WB analysis. Total cell lysates were also probed similarly (input). The experiments were repeated 3 times.

To further investigate whether Sap155 and *Sap190* are regulatory subunits of *Sit4* in *C. albicans*, we tested whether Sap155 and *Sap190* physically interact with *Sit4*. We tagged Sap155 and *Sap190* with GFP and *Sit4* with Flag all at the C-terminus. We then performed co-IP experiments using the anti-GFP-antibody conjugated beads to pull down Sap155-GFP and *Sap190*-GFP and then detected *Sit4*-Flag in western blotting analysis. The results showed that both Sap155 and *Sap190* physically associate with *Sit4* (Figure 1A). Furthermore, we tagged Sap155 C-terminus with Flag and *Sap190* C-terminus with GFP, pulled down *Sap190*-GFP, and then detected Sap155-Flag in western blotting analysis. We did not detect physical interaction between Sap155 and *Sap190* (Figure 1B). Thus, like *S. cerevisiae*, Sap155 and *Sap190* independently associate with *Sit4* in separate complexes in *C. albicans* (Luke et al., 1996).

### Characterization and Comparison of *sit4*Δ/Δ, *sap155*Δ/Δ, *sap190*Δ/Δ, and *sap155*Δ/Δ *sap190*Δ/Δ Mutants During Yeast Growth

To avoid the undesirable effects of having auxotrophic markers, we used the wild-type strain SC5314 as the parent and the *SAT1*-flipper method to delete the two copies of *SIT4*, *SAP155*, or *SAP190*, yielding the *sit4*Δ/Δ, *sap155*Δ/Δ, and *sap190*Δ/Δ mutants.

**FIGURE 2:**
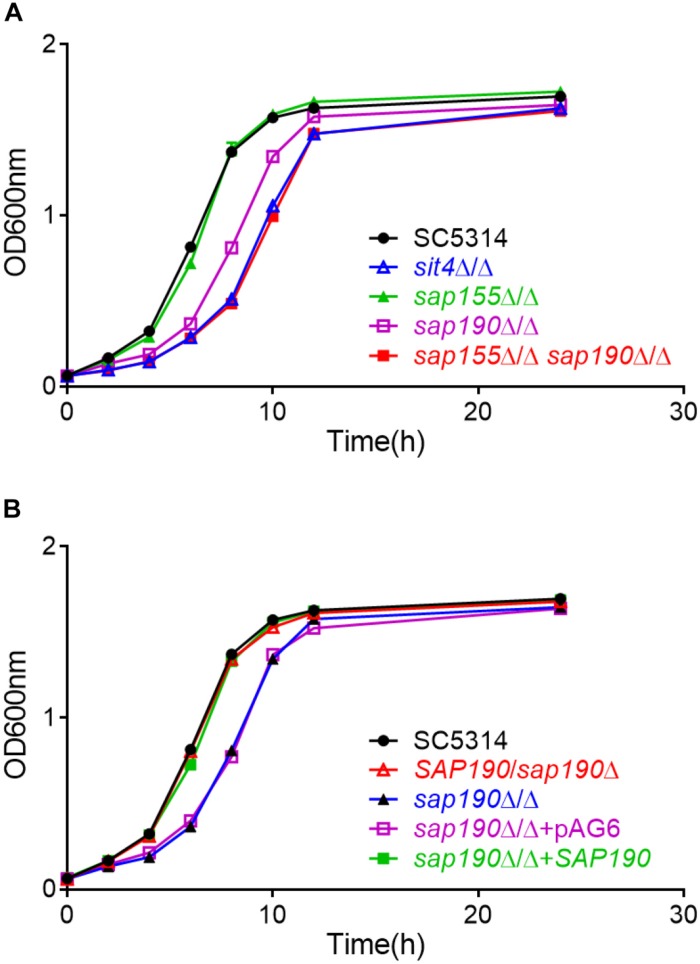
The growth of *Candida albicans* strains of the indicated genotype in YPD medium. Growth curves of **(A)** *C. albicans* SC5314, *sit4*Δ/Δ, *sap155*Δ/Δ, *sap190*Δ/Δ, and *sap155*Δ/Δ *sap190*Δ/Δ strains and **(B)** *C. albicans* SC5314, *SAP190*/*sap190*Δ, *sap190*Δ/Δ, *sap190*Δ/Δ + pAG6, and *sap190*Δ/Δ + *SAP190* strains in liquid YPD medium at 30^°^C with shaking at 200 rpm. All experiments were repeated using at least 3 independent clones for each mutant.

**FIGURE 3:**
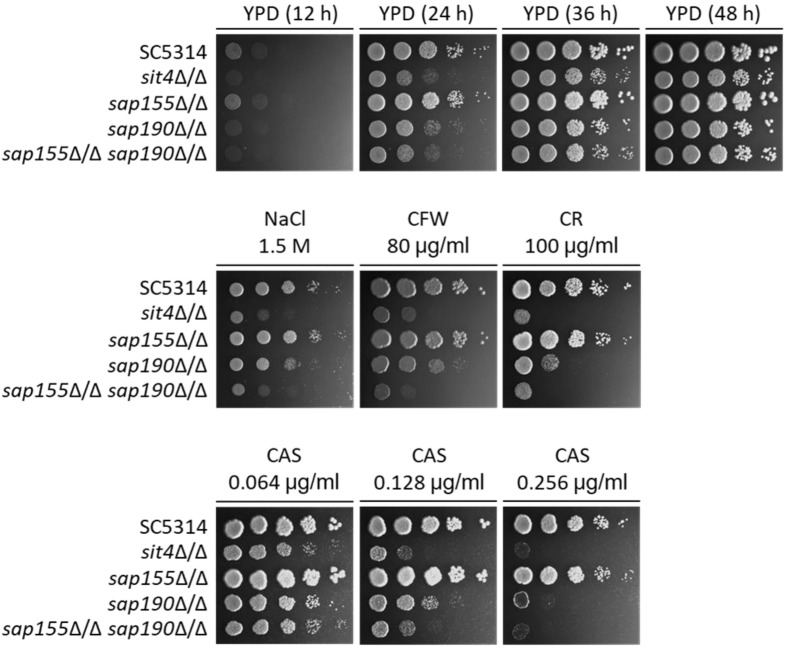
The sensitivity to several stress agents of *sit4*Δ/Δ, *sap155*Δ/Δ, *sap190*Δ/Δ, and *sap155*Δ/Δ *sap190*Δ/Δ mutants. Log-phase yeast cells of SC5314, *sit4*Δ/Δ, *sap155*Δ/Δ, *sap190*Δ/Δ, and *sap155*Δ/Δ *sap190*Δ/Δ strains were serially diluted 10-fold and spotted onto YPD plates, or YPD plates supplemented with 1.5 M NaCl, 80 μg/mL calcofluor white (CFW), 100 μg/mL congo red (CR), 0.064/0.128/0.256 μg/mL caspofungin (CAS). The YPD plates were incubated for the indicated times, and the YPD plates supplemented with stress agents were incubated at 30°C for 48 h. All experiments were repeated using at least 3 independent clones for each mutant.

To investigate the functions of the regulatory subunits of *Sit4* during yeast growth, wild-type (WT; SC5314), *sit4*Δ/Δ, *sap155*Δ/Δ, and *sap190*Δ/Δ cells were cultured in YPD liquid medium at 30°C and the growth was monitored by measuring OD_600_ at timed intervals (Figure 2A). Furthermore, yeast cultures of the same strains were serially diluted and spotted onto YPD plates (Figure 3). The results showed that the *sap155*Δ/Δ mutant exhibited normal growth, while the *sap190*Δ/Δ mutant grew much more slowly than WT cells. Introducing one copy of WT *SAP190* at the *RP10* locus of the *sap190*Δ/Δ mutant (*sap190*Δ/Δ + *SAP190*) fully restored the growth whereas introducing the empty vector pAG6 (*sap190*Δ/Δ + pAG6) had no effect, indicating that the slower growth of the *sap190*Δ/Δ mutant was due to the deletion of *SAP190* (Figure 2B). Also, the growth of the *sit4*Δ/Δ mutant was slightly slower than the *sap190*Δ/Δ mutant (Figures 2A, 3). To further determine the roles of *SAP155*, we deleted *SAP155* from the *sap190*Δ/Δ mutant to obtain the *sap155*Δ/Δ *sap190*Δ/Δ mutant. We found that the double mutant also grew little more slowly than the *sap190*Δ/Δ mutant while it grew at a similar rate to the *sit4*Δ/Δ mutant (Figures 2A, 3). These results suggest that *Sap190* is the main regulatory subunit of *Sit4* and is required for normal yeast growth of *C. albicans*, while *SAP155* can partially maintain cell growth in the absence of *SAP190*.

Next, we stained the nucleus with DAPI and found normal nuclear localization in *sit4*Δ/Δ, *sap155*Δ/Δ, *sap190*Δ/Δ, and *sap155*Δ/Δ *sap190*Δ/Δ cells (Supplementary Figure S2), and these mutants had normal cytoplasmic division, suggesting that the slow growth of cells lacking *SIT4* or its regulatory subunits is not due to abnormal cell division.

### Deletion of *SAP155* Renders the *sap190*Δ/Δ Mutant More Sensitive to Cell Wall Stress

A previous study has shown that the deletion of *SIT4* led to hypersensitivity to osmotic stress (Lee et al., 2004). We found that the *sap155*Δ/Δ *sap190*Δ/Δ and *sit4*Δ/Δ mutants grew at similar rates but both significantly more slowly than SC5314 (WT) on YPD plates containing 1.5 M NaCl (Figure 3). To investigate the functions of the regulatory subunits *SAP155* and *SAP190* in maintaining cell wall integrity, WT, *sit4*Δ/Δ, *sap155*Δ/Δ, and *sap190*Δ/Δ cells were spotted onto YPD plates containing 80 μg/ml Calcofluor White (CFW) or 100 μg/ml Congo Red (CR). We found that the growth of the *sap190*Δ/Δ mutant was slower than WT and the *sap155*Δ/Δ mutant, while the *sap155*Δ/Δ *sap190*Δ/Δ and *sit4*Δ/Δ mutants grew much more slowly than the *sap190*Δ/Δ mutant, and *sit4*Δ/Δ and *sap155*Δ/Δ *sap190*Δ/Δ mutants grew at similar rates (Figure 3). These results suggest that the deletion of *SAP155* further sensitizes the *sap190*Δ/Δ mutant to osmotic and cell wall stress.

The antifungal drug caspofungin (CAS) is a non-competitive inhibitor of β-1,3-glucan synthase and commonly used clinically to treat a variety of fungal infections, including *C. albicans* infections (Walker et al., 2010). We next determined whether lacking *Sit4* or its regulatory subunits also alters the sensitivity to CAS. We found that the sensitivity of *sap190*Δ/Δ mutant gradually increased with increasing concentrations of CAS in a range between 0.064 and 0.256 μg/ml and the deletion of *SAP155* in the *sap190*Δ/Δ mutant increased the sensitivity further, particularly at the concentration of 0.128 μg/ml (Figure 3). *sit4*Δ/Δ and *sap155*Δ/Δ *sap190*Δ/Δ mutants showed similar sensitivities to CAS under these conditions. These results indicate that *sit4*Δ/Δ and *sap190*Δ/Δ mutants are hypersensitive to CAS, consistent with their sensitivity to osmotic and cell wall stress. The results also show that *SAP155* is functional and can partially compensate for the absence of *SAP190*.

### The Functions of *SAP155* and *SAP190* in Filamentous Growth Caused by Different Inducing Factors

**FIGURE 4:**
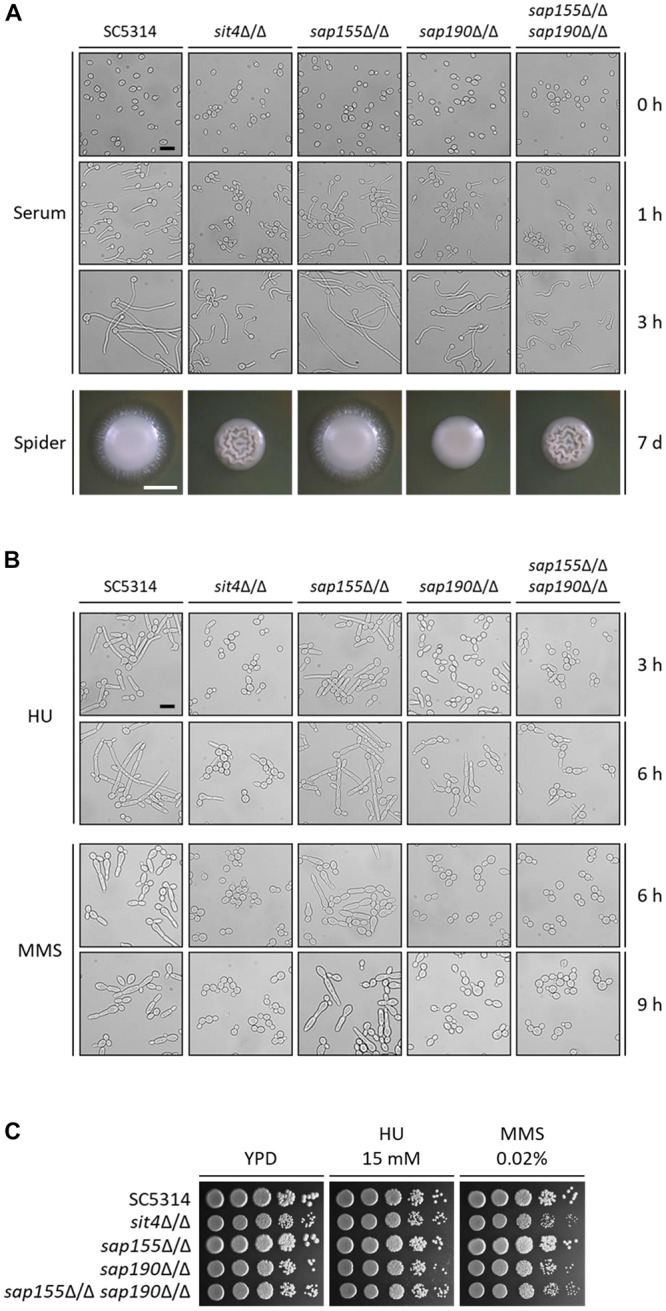
The morphogenesis of *sit4*Δ/Δ, *sap155*Δ/Δ, *sap190*Δ/Δ, and *sap155*Δ/Δ *sap190*Δ/Δ mutants. **(A)** Late-log phase yeast cells of SC5314, *sit4*Δ/Δ, *sap155*Δ/Δ, *sap190*Δ/Δ, and *sap155*Δ/Δ *sap190*Δ/Δ strains were re-inoculated at 1:20 dilution into fresh YPD medium containing 10% serum and incubated at 37°C with shaking at 200 rpm. Photos were taken at the indicated times. Size bars = 16 μm. Colony morphology of the same strains grown on Spider plates at 30^°^C for 2 or 7 days, respectively. Size bars = 1 mm. **(B)** Late-log phase yeast cells of the same strains as described in **(A)** were re-inoculated at 1:20 dilution into fresh YPD medium containing 15 mM HU or 0.02% MMS and incubated at 30°C with shaking at 200 rpm. Photos were taken at the indicated times. Size bars = 16 μm. **(C)** Log-phase yeast cells of the same strains as described in **(A)** were serially diluted 10-fold and spotted onto YPD plates supplemented with 15 mM hydroxyurea (HU) or 0.02% methyl methanesulfonate (MMS). The YPD plates were incubated at 30°C for 48 h.

We next examined the hyphal growth of the mutants of *SIT4* and its regulatory subunits under various inducing conditions. We found that although *sit4*Δ/Δ, *sap190*Δ/Δ, and *sap155*Δ/Δ *sap190*Δ/Δ cells could grow hyphae, their hyphae were much shorter compared with those of WT cells in YPD medium containing 10% serum at 37^°^C (Figure 4A). Interestingly, the hyphal length of *sap190*Δ/Δ cells was longer than that of *sit4*Δ/Δ and *sap155*Δ/Δ *sap190*Δ/Δ mutants. The same phenotype was also observed when hyphal growth was induced in DMEM medium (Supplementary Figure S3). *sap155*Δ/Δ cells did not exhibit significant defects in hyphal growth under these tested conditions. On Spider plates, WT and *sap155*Δ/Δ cells formed colonies with long filaments radiating from the colony periphery, while the edge of *sit4*Δ/Δ, *sap190*Δ/Δ, and *sap155*Δ/Δ *sap190*Δ/Δ colonies were smooth (Figure 4A). Furthermore, it is interesting that both *sit4*Δ/Δ and *sap155*Δ/Δ *sap190*Δ/Δ colonies show wrinkling at the colony center, which is very different from the morphology of the other strains. These results indicate that *SAP190* is required for hyphal growth and that *SAP155* can partially compensate for the loss of *SAP190* under some inducing conditions.

Genotoxic stress, such as DNA replication inhibition and DNA damage, can cause *C. albicans* cell cycle arrest, leading to filamentous growth and the formation of pseudohyphae (Gow et al., 2011). Hydroxyurea (HU) is an inhibitor of DNA replication, and MMS causes DNA methylation, leading to DNA damage. To investigate the roles of *Sit4* and its regulatory subunits in response to the genotoxic stress, we grew *C. albicans* in the presence of 15 mM HU and 0.02% MMS. We observed that, under HU treatment, while nearly all WT cells grew into long filaments, the majority of *sit4*Δ/Δ, *sap190*Δ/Δ, and *sap155*Δ/Δ *sap190*Δ/Δ cells remained in the yeast form with a small number of cells showing slight elongation (Figure 4B). The phenotypes of *sit4*Δ/Δ and *sap155*Δ/Δ *sap190*Δ/Δ cells were similar, both exhibiting more severe defects than *sap190*Δ/Δ cells. Under MMS treatment, *sit4*Δ/Δ, *sap190*Δ/Δ, and *sap155*Δ/Δ *sap190*Δ/Δ cells did not undergo filamentous growth (Figure 4B). After a 9-h MMS treatment, a small number of *sap190*Δ/Δ cells exhibited a slightly elongated yeast morphology, while all *sit4*Δ/Δ and *sap155*Δ/Δ *sap190*Δ/Δ cells remained in the typical yeast form. In spite of the defects in the genotoxic stress-induced filamentous growth, none of the mutants of *SIT4* and its regulatory subunits showed altered sensitivity to either HU or MMS (Figure 4C). These results indicate that, firstly, *Sit4* with its regulatory subunits plays an important role in regulating DNA-replication-inhibition and DNA-damage-induced filamentous growth. Secondly, *Sit4* and its regulatory subunits have different roles in filamentous growth in response to different types of genotoxic stress. Thirdly, *SAP190* plays a critical role in the filamentous growth induced by genotoxic stress, while *SAP155* partially compensates for the loss of *SAP190*.

### Characterization and Comparison of the Virulence of *sit4*Δ/Δ, *sap155*Δ/Δ, *sap190*Δ/Δ, and *sap155*Δ/Δ *sap190*Δ/Δ Mutants *in vitro* and *in viv*o

**FIGURE 5:**
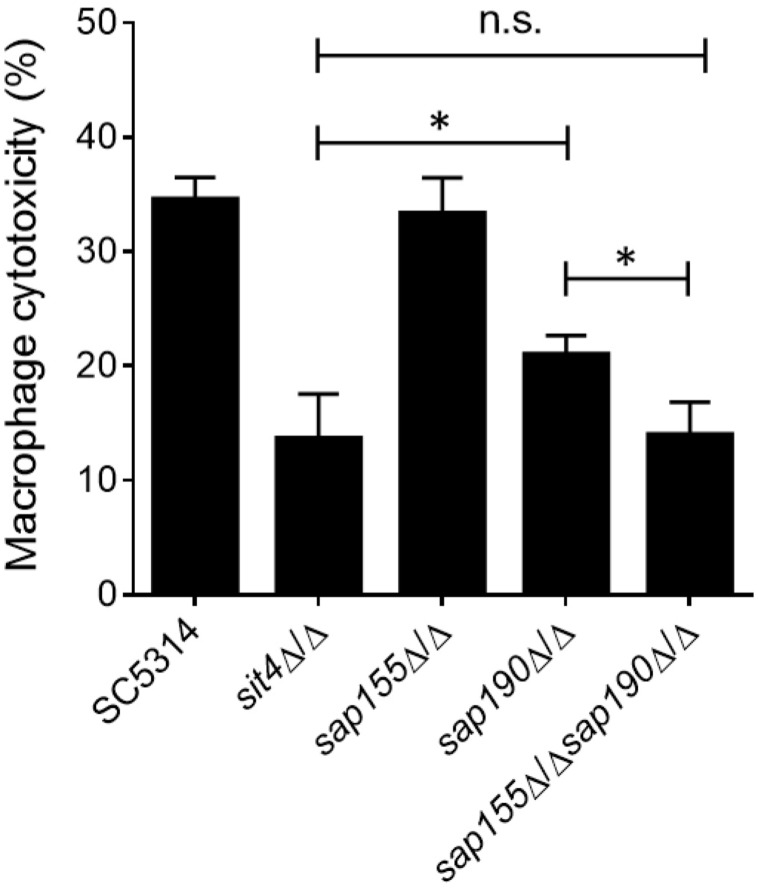
The ability of *sit4*Δ/Δ, *sap155*Δ/Δ, *sap190*Δ/Δ, and *sap155*Δ/Δ *sap190*Δ/Δ mutants to damage macrophages *in vitro*. Yeast cultures of SC5314, *sit4*Δ/Δ, *sap155*Δ/Δ, *sap190*Δ/Δ, and *sap155*Δ/Δ *sap190*Δ/Δ strains were co-cultured with RAW264.7 macrophages for 5 h. Cytotoxicity was determined using an LDH release assay. Results are the mean ± SD of three independent experiments, each performed in triplicates. The results were analyzed with Student’s *t*-test. ^∗^*p* < 0.05 compared between the indicated two groups. n.s.: no significance.

Macrophages are the first line of host defense against *C. albicans* infection, but *C. albicans* can escape through its hyphal growth, which can penetrate and cause the lysis of macrophages (Erwig and Gow, 2016). Next, we co-cultured *C. albicans* with RAW264.7 macrophages and then measured the activity of lactate dehydrogenase (LDH) released by macrophages into the supernatant to determine the macrophage cytotoxicity of *C. albicans.* We found that cells lacking *SAP155* did not alter the macrophage cytotoxicity, while cells lacking *SAP190* resulted in less damage to macrophages (Figure 5). Furthermore, *sap155*Δ/Δ *sap190*Δ/Δ and *sit4*Δ/Δ mutants exhibited similar macrophage cytotoxicity while both caused less damage to macrophages than the *sap190*Δ/Δ mutant.

**FIGURE 6:**
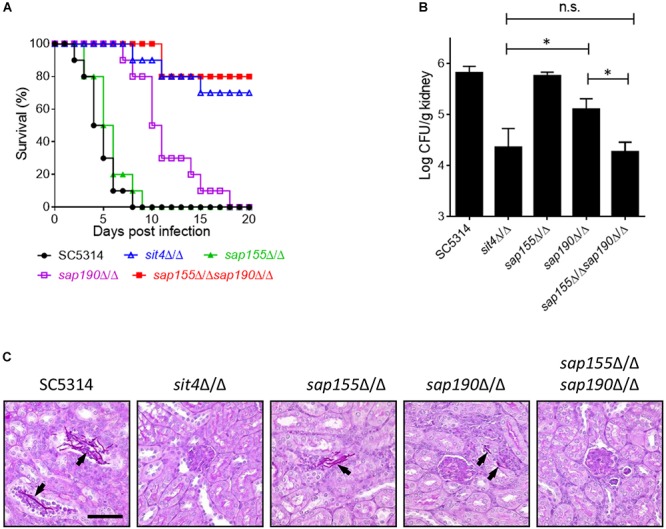
The virulence of *sit4*Δ/Δ, *sap155*Δ/Δ, *sap190*Δ/Δ, and *sap155*Δ/Δ *sap190*Δ/Δ mutants in a murine model of disseminated candidiasis BALB/c mice were injected via the tail vein with 10^6^ yeast cells of SC5314, *sit4*Δ/Δ, *sap155*Δ/Δ, *sap190*Δ/Δ, or *sap155*Δ/Δ *sap190*Δ/Δ strains (15 mice per *C. albicans* strain) and monitored for **(A)** survival over a period of 20 days. The results of survival curves were analyzed using Kaplan-Meier test. The statistical analysis showed that the survival curves of *sap190*Δ/Δ strain are significantly different compared to SC5314 or *sap155*Δ/Δ strain, and the survival curves of *sit4*Δ/Δ or *sap155*Δ/Δ *sap190*Δ/Δ strain are significantly different compared to SC5314, *sap155*Δ/Δ, or *sap190*Δ/Δ strain. Five mice were sacrificed after 48 h of the injection to **(B)** determine the fungal load in the kidney (the results of fungal burdens were analyzed with Mann-Whitney test. ^∗^*p* < 0.05 compared between the indicated two groups. n.s: no significance) and **(C)** conduct histological examinations of kidney sections. Size bars = 0.13 mm. Arrows indicate *C. albicans* cells in the renal tissues.

We next investigated the role of *Sit4* and its regulatory subunits in virulence using a mouse model of systemic infection. Mice were injected with the WT (SC5314), *sit4*Δ/Δ, *sap155*Δ/Δ, *sap190*Δ/Δ, or *sap155*Δ/Δ *sap190*Δ/Δ strains via the tail vein, and their survival was monitored for 20 days. The results showed that all mice injected with WT or *sap155*Δ/Δ strains died within 9 days, and their survival median was 4–6 days. Mice infected with the *sap190*Δ/Δ mutant all died within 18 days, and the survival median was 10–11 days. However, ≥50% of mice injected with the *sit4*Δ/Δ or s*ap155*Δ/Δ *sap190*Δ/Δ mutant survived for at least 20 days (Figure 6A). To assess the ability of these mutants to colonize the kidney, Five mice in each group were sacrificed 48 h post-infection to quantify CFUs and perform PAS staining of kidney sections. The results showed that CFUs in the kidneys of mice infected with the *sit4*Δ/Δ, *sap190*Δ/Δ, or *sap155*Δ/Δ *sap190*Δ/Δ mutant were much less than that in mice infected with the WT strain or the *sap155*Δ/Δ mutant. There was no statistically significant difference between the CFUs of *sit4*Δ/Δ and *sap155*Δ/Δ *sap190*Δ/Δ mutants, but the CFUs of both mutants were marked more than that of the *sap190*Δ/Δ mutant (Figure 6B). Also, the PAS staining of kidney sections revealed many long filaments of *C. albicans* cells in mice injected with SC5314 or the *sap155*Δ/Δ strain. In contrast, a small number of *C. albicans* cells were found in the kidney of mice infected with the *sap190*Δ/Δ mutant, and none was found in the kidney of mice infected with the *sit4*Δ/Δ or *sap155*Δ/Δ *sap190*Δ/Δ mutant (Figure 6C). The results demonstrate that *SAP190* is required for the virulence of *C. albicans* and that *SAP155* can partially compensate for the loss of virulence in the absence of *SAP190* during co-cultivation with macrophages and systemic infection of mice.

## Discussion

PP2A-like phosphatases share high homology with PP2A enzymes which contain a catalytic subunit and a regulatory subunit (Albataineh and Kadosh, 2016). *C. albicans Sit4* has been identified as a PP2A-like catalytic subunit (Lee et al., 2004). In this study, according to the amino-acid sequence analyses, we identified two proteins in *C. albicans* encoded by orf19.642 and orf19.5160 which are homologous to *Sit4* regulatory subunits SAPs in *S. cerevisiae*. They share the relatively high homology of 26.5% and 35.0% with ScSap155 and Sc*Sap190*, respectively. Furthermore, co-IP experiments showed that like in *S. cerevisiae*, both proteins physically associate with *Sit4*, forming separate complexes in *C. albicans* (Luke et al., 1996). We show here that *Sap190* is the main regulatory subunit of *Sit4* and plays critical roles in cell growth, cell wall integrity, morphogenesis, and virulence in mice. In the SC5314 background, deleting *SAP155* does not produce any apparent defects, but deleting it in the *sap190*Δ/Δ background leads to more severe defects, indicating that Sap155 is functionally redundant and can partially compensate for the absence of *Sap190*. These findings also indicate that *C. albicans* retains redundant regulatory subunits of *Sit4*, which may enhance its adaptability to some adverse environmental factors.

Cytokinesis and nuclear localization in *sit4*Δ/Δ, *sap155*Δ/Δ, *sap190*Δ/Δ, and *sap155*Δ/Δ *sap190*Δ/Δ mutants do not exhibit any discernable defects. In *S. cerevisiae*, *SIT4* is required for the G1/S transition (Sutton et al., 1991), and in *Debaryomyces hansenii*, deletion of *SIT4* causes an increased number of G1 phase cells (Chawla et al., 2017). Therefore, like in other fungal species, the regulatory subunits of *Sit4* may affect cell growth though the G1/S transition in *C. albicans*.

The yeast-to-hyphae morphological transition is recognized as the most important trait for *C. albicans* infection. Previous studies have shown that *SIT4* is involved in the morphogenesis of *C. albicans* (Lee et al., 2004; Noble et al., 2010). Our results show that *sap190*Δ/Δ cells exhibited slower hyphal formation with shorter hyphal length, and *sap155*Δ/Δ *sap190*Δ/Δ cells exhibit more severe defects, which was similar to *sit4*Δ/Δ mutant, although the *sap155*Δ/Δ mutant did not show any discernible defects. The results suggest that both regulatory subunits of *Sit4* are involved in regulating morphogenesis. In *C. albicans*, cell wall integrity is closely correlated with morphogenesis. For example, the deletion of cell wall protein-coding genes *ECM33* and *CSF4* led to abnormal hyphal growth (Alberti-Segui et al., 2004; Martinez-Lopez et al., 2004). Thus, *SAP155* and *SAP190* may regulate morphogenesis partially through their roles in cell growth and cell wall integrity. However, the targets of *SIT4* and its regulatory subunits are remain unclear in *C. albicans*, thus their roles in the morphogenesis may be also through other unknown mechanisms.

Genotoxic stress, such as DNA replication inhibition and DNA damage, can activate cell cycle checkpoints via the phosphorylation of the checkpoint protein kinase Rad53, causing *C. albicans* to form pseudohyphae (Gow et al., 2011). Deletion of *RAD53* not only led to a defect in filamentous growth, but also caused hypersensitivity to genotoxic stress (Shi et al., 2007). Interestingly, we show here that under HU treatment, only a small percentage of *sit4*Δ/Δ, *sap190*Δ/Δ, and *sap155*Δ/Δ *sap190*Δ/Δ cells could undergo filamentous growth forming short filaments, and under MMS treatment, these mutant cells remained in the yeast form. However, deletion of *SIT4* or its regulatory subunits did not alter the sensitivity to HU and MMS, suggesting that *Sit4* with its regulatory subunits are involved in DNA-replication and DNA-damage checkpoint pathways to specifically regulate the filamentous growth. In future studies, we will explore the relationship between *SIT4* phosphatase complexes and Rad53 under different genotoxic stresses.

Although the exact mechanism by which Sap155 and *Sap190* regulate the functions of *Sit4* remains unclear, deleting *SAP190* causes reduced macrophage cytotoxicity *in vitro* and impaired virulence in mice, and deleting *SAP155* in *sap190*Δ/Δ background resulted in more severe defects. Therefore, the regulatory subunits of *Sit4* could serve as targets for developing new antifungal drugs.

## Data Availability Statement

The datasets generated for this study are available on request to the corresponding author.

## Ethics Statement

The animal experiments were carried out in accordance to National Advisory Committee for Laboratory Animal Research Guidelines, and all procedures were approved by the IACUC of the Agency for Science, Technology and Research of Singapore.

## Author Contributions

QH, JS, and YW conceived and created the experimental design. QH, CP, and YQW conducted the experiments. QH, YW, and LZ prepared the manuscript.

## Conflict of Interest

The authors declare that the research was conducted in the absence of any commercial or financial relationships that could be construed as a potential conflict of interest.

## Footnotes

1 http://blast.ncbi.nlm.nih.gov/Blast.cgi

## Supplementary Material

FIGURE S1 Sequence alignment of *S. cerevisiae Sap155* and *Candida albicans Sap155* (orf19.642), *S. cerevisiae Sap190* and *C. albicans Sap190* (orf19.5160) Completely conserved residues are colored cyan.

FIGURE S2 The location of nucleus in *sit4*Δ/Δ, *sap155*Δ/Δ, *sap190*Δ/Δ, and *sap155*Δ/Δ *sap190*Δ/Δ cells. Yeast cells of *C. albicans* strains of the indicated genotype were stained with DAPI to visualize the nucleus. Size bars = 12 μm.

FIGURE S3 The hyphal growth of *sit4*Δ/Δ, *sap155*Δ/Δ, *sap190*Δ/Δ, and *sap155*Δ/Δ *sap190*Δ/Δ mutants in DMEM. Late-log phase yeast cells of SC5314, *sap155*Δ/Δ, *sap190*Δ/Δ, and *sap155*Δ/Δ *sap190*Δ/Δ strains were re-inoculated at 1:20 dilution into fresh DMEM and incubated at 37°C with shaking at 200 rpm. Photos were taken at 1 h and 3 h. Size bars = 16 μm.
