# Supplementary figures and images for: PP2A-Like Protein Phosphatase (Sit4) Regulatory Subunits, Sap155 and Sap190, Regulate Candida albicans’ Cell Growth, Morphogenesis, and Virulence
Source: Front Microbiol. 2019 Dec 20;10:2943. doi: 10.3389/fmicb.2019.02943 (PMC6933005; doi:10.3389/fmicb.2019.02943)

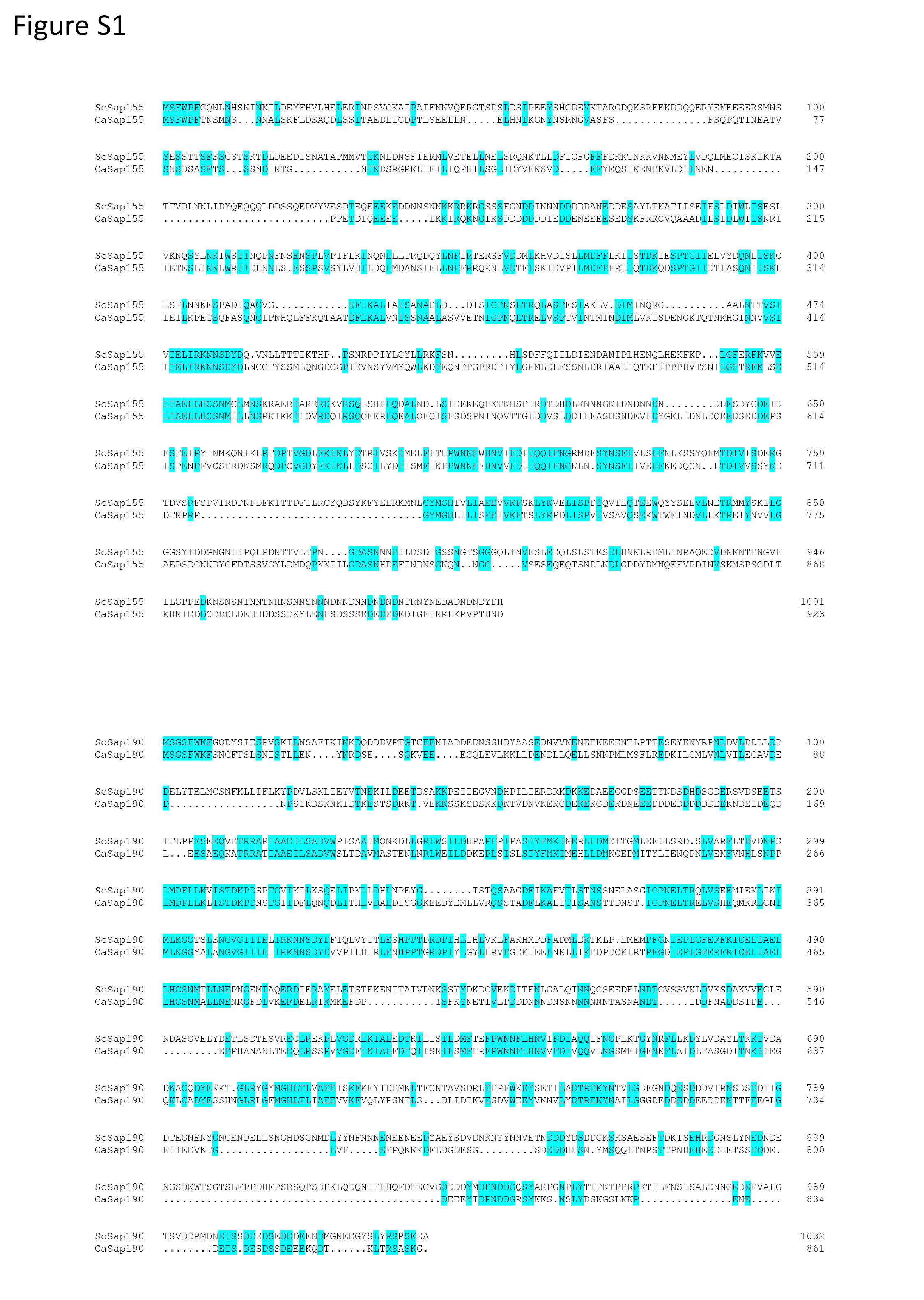

Supplement: FIGURE S1 — Sequence alignment of S. cerevisiae Sap155 and Candida albicans Sap155 (orf19.642), S. cerevisiae Sap190 and C. albicans Sap190 (orf19.5160) Completely conserved residues are colored cyan. [file Image_1.JPEG]

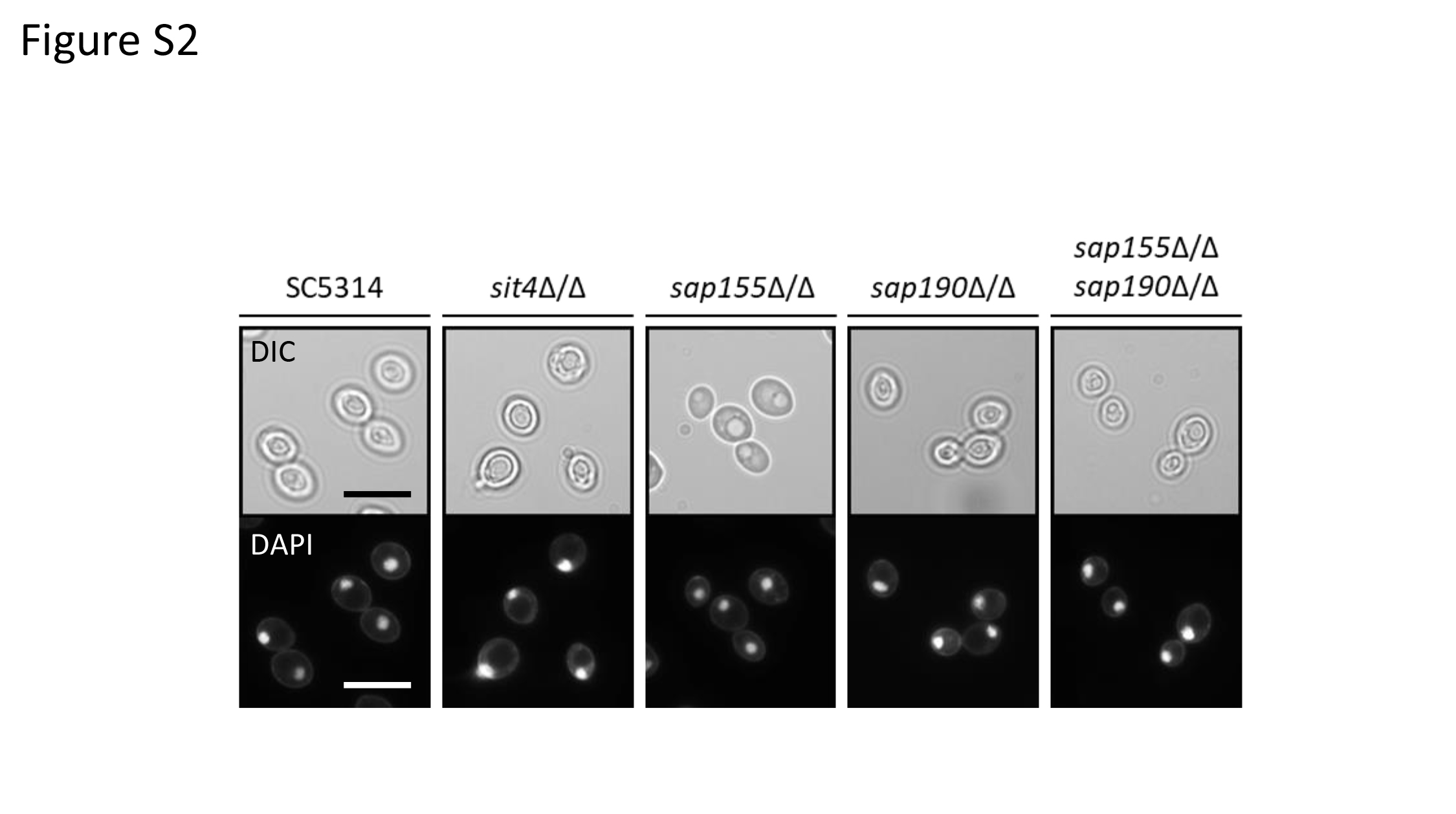

Supplement: FIGURE S2 — The location of nucleus in sit4Δ/Δ, sap155Δ/Δ, sap190Δ/Δ, and sap155Δ/Δ sap190Δ/Δ cells. Yeast cells of C. albicans strains of the indicated genotype were stained with DAPI to visualize the nucleus. Size bars = 12 μm. [file Image_2.JPEG]

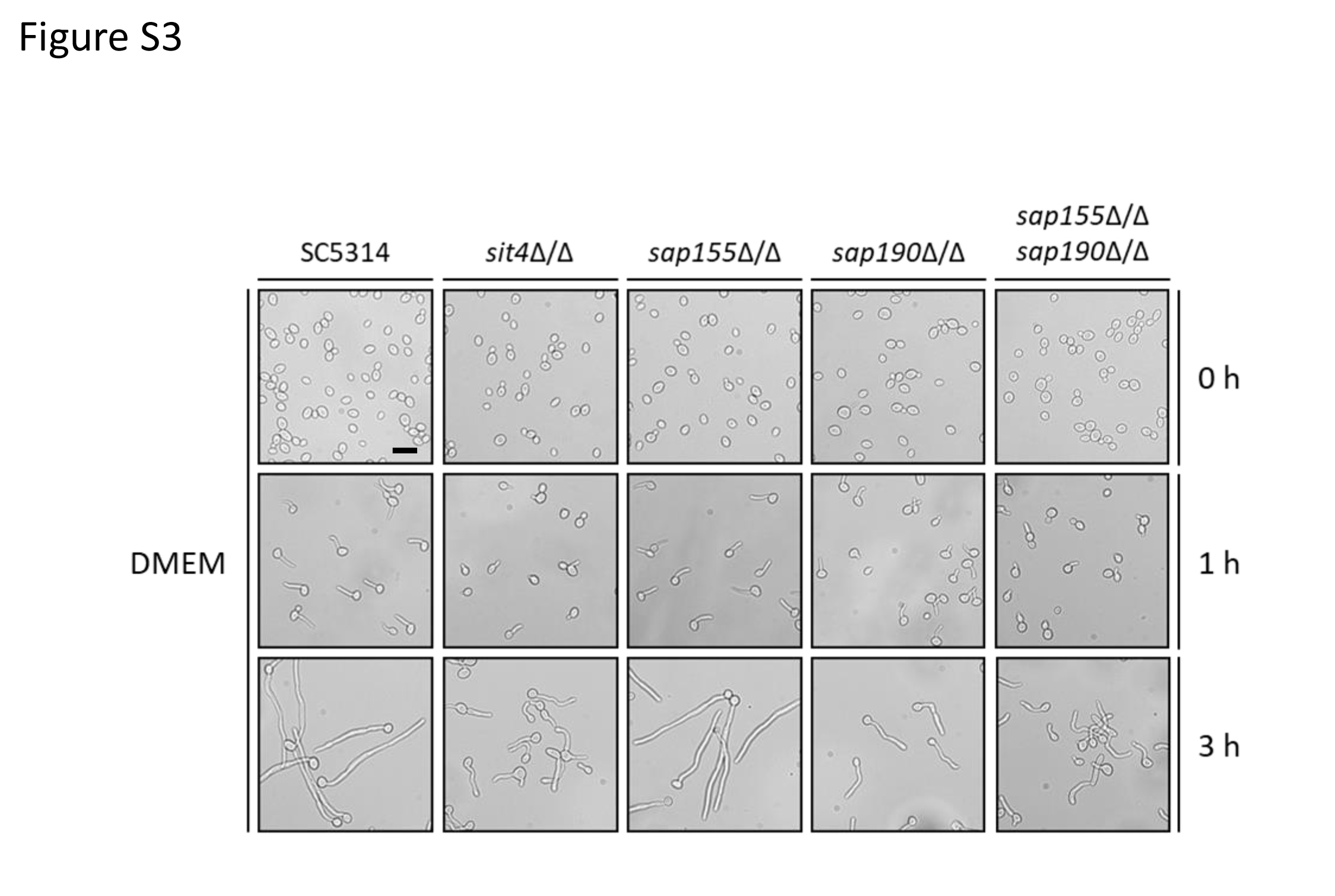

Supplement: FIGURE S3 — The hyphal growth of sit4Δ/Δ, sap155Δ/Δ, sap190Δ/Δ, and sap155Δ/Δ sap190Δ/Δ mutants in DMEM. Late-log phase yeast cells of SC5314, sap155Δ/Δ, sap190Δ/Δ, and sap155Δ/Δ sap190Δ/Δ strains were re-inoculated at 1:20 dilution into fresh DMEM and incubated at 37°C with shaking at 200 rpm. Photos were taken at 1 h and 3 h. Size bars = 16 μm. [file Image_3.JPEG]
